# Stress-Activated Protein Kinases in Spinal Cord Injury: Focus on Roles of p38

**DOI:** 10.3390/ijms19030867

**Published:** 2018-03-15

**Authors:** Yoshitoshi Kasuya, Hiroki Umezawa, Masahiko Hatano

**Affiliations:** 1 Department of Biomedical Science, Graduate School of Medicine, Chiba University, Chiba City, Chiba 260-8670, Japan; h-umepan@hotmail.co.jp (H.U.); hatanom@faculty.chiba-u.jp (M.H.); 2 Department of Biochemistry and Molecular Pharmacology, Graduate School of Medicine, Chiba University, Chiba City, Chiba 260-8670, Japan; 3 Department of Respirology, Graduate School of Medicine, Chiba University, Chiba City, Chiba 260-8670, Japan

**Keywords:** spinal cord injury, stress-activated protein kinases, c-Jun N-terminal kinase, p38 mitogen-activated protein kinase

## Abstract

Spinal cord injury (SCI) consists of three phases—acute, secondary, and chronic damages—and limiting the development of secondary damage possibly improves functional recovery after SCI. A major component of the secondary phase of SCI is regarded as inflammation-triggered events: induction of cytokines, edema, microglial activation, apoptosis of cells including oligodendrocytes and neurons, demyelination, formation of the astrocytic scar, and so on. Two major stress-activated protein kinases (SAPKs)—c-Jun N-terminal kinase (JNK) and p38 mitogen-activated protein kinase (p38 MAPK)—are activated in various types of cells in response to cellular stresses such as apoptotic stimuli and inflammatory waves. In animal models of SCI, inhibition of either JNK or p38 has been shown to promote neuroprotection-associated functional recovery. Here, we provide an overview on the roles of SAPKs in SCI and, in particular, the pathological role of p38 will be discussed as a promising target for therapeutic intervention in SCI.

## 1. Introduction

Traumatic spinal cord injury (SCI) is a severe, devastating disease which often results in sensorimotor dysfunction, largely due to the poor regenerative capacity of neuronal cells in the adult mammalian central nervous system (CNS) [1]. Following the initial physical injury, the lesion proceeds to receive secondary waves of complex neuroinflammatory events that cause additional tissue destruction and functional impairments [2,3]. Under the secondary phase of SCI, however, gradual functional recovery is observed in not only several animal models but also humans to some extent, and is inversely proportional to the initial damage intensity of the SC [4]. In developing beneficial drugs for SCI, it is therefore clinically reasonable to target the secondary damages involving inflammation, blood–cerebrospinal fluid (CSF) barrier disruption, glial scar formation, cell death of neurons and oligodendrocytes, lipid peroxidation, and glutamate excitotoxicity. Indeed, many studies have recently focused on pharmacological interventions targeting the secondary damage symptoms. To strengthen the reliability of therapeutic candidates for SCI, however, the exact molecular mechanism underlying the secondary damage symptoms and the pivotal signaling pathway mediating each symptom should be evaluated precisely.

The stress-activated protein kinase (SAPK) group of mitogen-activated protein kinases (MAPKs) includes two members of the c-Jun NH2-terminal kinase (JNK) and p38 MAPK families, which are activated in response to environmental stresses such as inflammatory stimuli by cytokines and Toll-like receptor (TLR) ligands, oxidative stress, trophic factor withdrawal, osmolarity shock, ultraviolet (UV) irradiation, chemotherapeutic drugs, and so on [5,6]. The JNK family (JNK1, JNK2, and JNK3) are encoded by three separate but closely related genes. JNK1 and JNK2 are expressed ubiquitously in adult tissues, whereas the expression of JNK3 is primarily observed in brain, heart, and testis. Ten different JNK isoforms are produced by alternative splicing of the *Jnk* transcripts: JNK1α1, JNK1β1, JNK2α1, JNK2β1, and JNK3α1 have a molecular weight of 46 kDa; and JNK1α2, JNK1β2, JNK2α2, JNK2β2, and JNK3α2 have a molecular weight of 54 kDa with an extended C-terminus [7]. Their relative contributions to the overall JNK activity remain to be elucidated. JNKs are activated by dual phosphorylation of the TPY motif within their activation loop by two upstream MAPK kinases (MAP2Ks)—MKK4 and MKK7—which are activated by various MAPKK kinases (MAP3Ks), MEKKs, Mixed-lineage kinases (MLKs), apoptosis signal-regulating kinase 1 (ASK1), thousand-and-one amino acid kinase 2 (TAO2), TNF receptor-associated factor 2- and NCK-interacting protein kinase (TNIK), and dual leucine zipper-bearing kinase (DLK) [8].

On the other hand, the p38 family consists of four isoforms (α, β, γ, and δ) arising from separate genes. p38α and -β are ubiquitously expressed in adult tissues, whereas expression of p38γ is predominant in skeletal muscle and p38δ shows high expression levels in the kidney and lung [9,10]. As an alternative form of p38β that was initially identified, p38β2 with an internal deletion of 8 amino acids has been reported. p38β2 showed much higher sensitivity to extracellular stimuli and p38 inhibitor than does p38β, which shows a level similar to that of p38α. In particular, p38β2 but not p38β phosphorylated various substrates (as p38α does) in response to sorbitol [11]. Therefore, p38β means p38β2 at present. Among p38 isoforms, the best characterized isoform is p38α, the physiological and pathological roles of which have been well investigated [5,12]. In this review, we therefore mostly refer to p38α as p38, unless otherwise indicated. p38 MAPKs are activated by dual phosphorylation of the TGY motif within their activation loop by two upstream MAP2K—MKK3 and MKK6—which are activated by various MAP3Ks, MEKKs, MLKs, ASK1, TAO2, TGF-β-activated kinase 1 (TAK1), and Tumor progression locus 2 (TPL2). Consequently, JNK and p38 pathways share a number of upstream MAP3Ks although the two pathways are not redundant. In addition to this canonical activation pathway composed of three stepwise modules, specific binding of TAK1-binding protein 1 (TAB1) to p38α and TCR/ζ chain-associated protein kinase (ZAP70)-mediated phosphorylation of Tyr^323^ in the C-terminal domain of p38 MAPKs (except p38δ) are described as new p38 activation pathways via upregulating autophosphorylation of p38 MAPKs [13,14].

**Figure 1:**
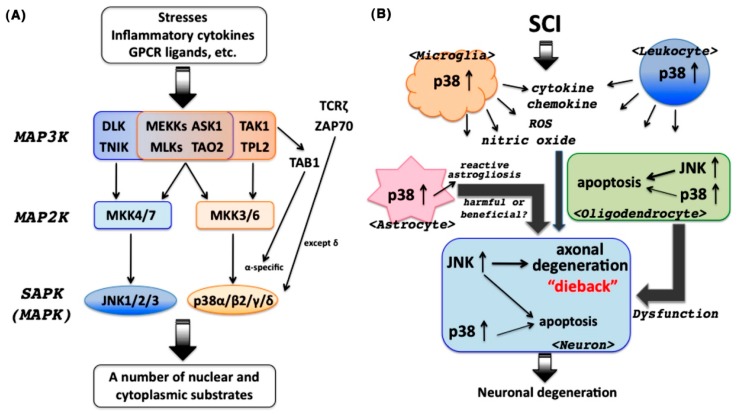
(**A**) Overview of the stress-activated protein kinase (SAPK) pathway. SAPKs, c-Jun N-terminal kinases (JNKs), and p38 mitogen-activated protein kinases (MAPKs) are activated in response to a variety of cellular stresses through a three-step pathway (MAP3K/MAP2K/MAPK). In addition to this canonical pathway, several pathways for p38 activation have been demonstrated. (**B**) Overview of the SAPK-mediated neuronal degeneration after spinal cord injury (SCI). JNK contributes to neuronal degeneration in a direct manner and also induces neuronal dysfunction in an indirect manner through oligodendrocytic cell-death-associated demyelination. p38 predominantly orchestrates SCI-triggered inflammatory responses such as activation of microglia, production of inflammatory and neurotoxic mediators from infiltrated leukocytes and activated microglia, and reactive astrogliosis. Reactive astrogliosis shows bidirectional effects on neuronal regeneration after SCI.

Overview of the SAPK activation pathway is shown in Figure 1A. SAPKs activated through a typical kinase cascade promote a variety of cellular responses. In this review, we introduce increasing evidence concerning pathological functions of JNK and p38 in SCI. In particular, we will discuss the potential of targeting the p38 pathway as a disease-modifying therapy in SCI.

## 2. SAPKs in the CNS

In the CNS, JNK1 and JNK2 are expressed in various types of cells. On the other hand, the expression of JNK3 is predominantly observed in neuronal cells. The most highly expressed transcript of JNK isoform in the adult rodent brain is *Jnk3* mRNA followed by *Jnk2* mRNA and then *Jnk1* mRNA [15,16]. The cellular and behavioral phenotypes observed in *JNK* isoform- and compound *JNK* isoforms-knockout mice models clearly suggest crucial roles of JNKs in the CNS as follows: (i) *Jnk3*-knockout mice exhibit a marked reduction in kainate-induced neuronal apoptosis in the hippocampus secondary to seizure response, and in dopaminergic cell loss in the Parkinson’s disease model mice using 1-methyl-4-phenyl-1,2,4,6-tetrahydropyridine (MPTP) [17,18,19]; (ii) *Jnk2*-knockout mice show a decrease in dopaminergic cell loss in the MPTP Parkinson’s disease model [18]; (iii) *Jnk1/Jnk2*-double-knockout mice show an embryonic lethality at E11.5 because of severe dysregulation of apoptosis in the hindbrain at E9.0 [20]; (iv) *Jnk1/Jnk2/Jnk3*-triple-knockout neuronal cells (primary cultured neurons from *Jnk1^LoxP/LoxP^Jnk2^−/−^Jnk3^−/−^* mice were infected with *Ad-cre* to establish deficiency of the *JNKs*) exhibit marked life span extension during culture in vitro [21]. Therefore, JNK is the central player at least in neuronal apoptosis, which tempts us to consider that JNKs are able to contribute to the development of various neural diseases. Here, “knockout” represents a traditional gene knockout, unless otherwise indicated.

In contrast to the cases of *JNK*-knockout mice, deficiency of each p38 isoform gene does not result in defects in the CNS. *p38α*-knockout mice result in embryonic lethality due to dysfunction of erythropoiesis and placental organogenesis [22,23]. A deficiency of the *p38β* gene is functionally compensated by p38 in both activation of downstream kinases and TNF-α-mediated inflammatory diseases [24]. *p38γ*-knockout mice exhibit loss of myogenic precursor cells that are primarily responsible for skeletal muscle growth and regeneration [25]. *p38δ*-knockout mice exhibit a marked resistance to skin tumor development induced by chemical agents [26]. However, increasing evidence indicates that at least p38α is expressed in neurons, astrocytes, oligodendrocytes, and microglia and controls their cell fate and functions [12,27,28,29,30]. For instance, it has been proposed that amyloid β-peptide (Aβ)-induced disruption of N-cadherin-based synaptic contact results in p38 activation, which in turn phosphorylates Tau leading to neuronal apoptosis. This phenomenon is one of the neurodegenerative processes in Alzheimer’s disease (AD) [31]. Likewise, p38 as well as JNK are activated and play roles in the innate and adaptive immune responses, and various p38 inhibitors have been energetically developed and are in clinical trials as potential anti-inflammatory drugs [5] (Clinicaltrials.gov, https://clinicaltrials.gov/). In inflammation-associated disorders of the CNS, p38 may therefore be a valid therapeutic target. In fact, several reports including our study have demonstrated that p38α contributes to the pathogenesis of experimental autoimmune encephalomyelitis (EAE, a model for multiple sclerosis) mainly via mobilizing the Th17/IL-17 axis [32,33,34,35,36]. Notably, p38 inhibitors ameliorate the progression of EAE if administered even after the onset of clinical symptoms [35,36]. In AD, moreover, the intense signal of p38 activation was observed in the brain area where neuritic β-amyloid plaques were accumulated. It has been also demonstrated that p38 plays a crucial role in amyloid precursor protein-induced production of neurotoxic cytokines (e.g., TNF-α and IL-1β) in microglia [37]. It is noteworthy that p38α inhibition ameliorates the overproduction of proinflammatory cytokines associated with neuronal dysfunction and behavioral deficits in the Aβ-infused AD-relevant animal model [38].

## 3. Involvement of JNK in the Pathogenesis of SCI

Early evidence has demonstrated that contusion SCI activates the ASK1/JNK-p38 signaling axis in both neurons and oligodendrocytes proceeding to apoptosis [39]. These events were followed by further study using tiptoe-walking Yoshimura (TWY) mice that developed aging-associated spontaneous calcification in the cervical ligament, thereby causing chronic mechanical compression of the spinal cords. [40]. In those reports, the activation of ASK1-JNK and -p38 pathways was observed in apoptotic sign-bearing neurons and oligodendrocytes, suggesting that JNK and p38 contribute to neuronal and oligodendrocytic apoptosis in SCI at least phenomenologically.

The JNK-mediated apoptosis of oligodendrocytes in SCI has been precisely investigated [41]. The injury-activated JNK3 phosphorylated myeloid cell leukemia sequence-1 (Mcl-1) and facilitated the degradation of Mcl-1 by ubiquitination in oligodendrocytes, which in turn induced apoptosis-associated release of cytochrome C from mitochondria. This JNK-mediated oligodendrocytic apoptosis was canceled by a disruption of the *Jnk3* gene. Interestingly, the peak activation ratio of JNK3 was 500-fold higher than those of JNK1/2 in the SC after hemisection injury, and JNK activation monitored by phosphorylation of c-Jun was typically detected in neurons as well. Nevertheless, the number of apoptotic neuron somas (TUNEL^+^/NeuN^+^) was not significantly different between *Jnk3*-knockout mice and wild-type (WT) littermates, suggesting several possibilities as follows: (i) low activation of JNK1 or JNK2 functionally contributes to neuronal cell death after SCI; (ii) JNK3 may induce not apoptosis but another type of cell death—autophagic death—after SCI; (iii) JNK3 may not directly regulate apoptosis of neurons but may play a role in transporting the damage signal under nerve degeneration or regeneration like the case in the previous report [42]. The last notion was supported by a subsequent study.

Superior cervical ganglion 10 (SCG10), an axonal-maintenance factor, has phosphorylation sites (Ser63 and Ser73) specific for JNK and is at least partly regulated by the phosphorylation-triggered degradation program. In healthy axons, SCG10 protein may undergo rapid JNK-dependent degradation versus replenishment by axonal transport from cell bodies. Once axons were injured, loss of function of the replenishment in concert with continuous JNK-dependent degradation might result in a marked loss of SCG10 selectively in distal axon segments, which accelerates axonal fragmentation leading to anterograde axonal degeneration [43]. Hence, the JNK/SCG10 axis may contribute to Wallerian degeneration, one of the typical pathological aspects in SCI. Furthermore, the involvement of JNK in retrograde axonal degeneration—so-called “dieback”—has been clearly demonstrated [44]. The SCI-upregulated phospho-JNK was observed in the dorsal corticospinal tract (CST) fibers labeled with biotinylated dextran amine (BDA) in the gray matter rostral to the lesion site, where amyloid-β precursor protein, a marker for axonal degradation, was apparently accumulated. This SCI-induced axonal dieback was markedly suppressed by continuous intrathecal administration of a JNK inhibitor, SP600125, which was replicated in both *JNK1*-knockout and *JNK3*-knockout mice. Likewise, hindlimb locomotor recovery after SCI was improved in SP600125-treated WT, *JNK1*-knockout, and *JNK3*-knockout mice, suggesting that at least JNK1 and JNK3 positively regulate axonal dieback limiting locomotor recovery after SCI. It has been also demonstrated that the improvement of functional recovery after SCI is performed by D-JNKI1—the cell-permeable peptide inhibitor against JNK [45]. Notably, a single intraperitoneal injection of D-JNKI1 showed beneficial effects on SCI, which may be valuable as a minimally invasive strategy for clinical application of JNK inhibition.

In contrast to the axonal degeneration activity of JNK in SCI, a lot of previous reports have demonstrated that JNKs might play important roles in axonal guidance and neurite growth [8]. During the axonal regeneration process, JNK activity is necessary for neuritogenesis and sustained neurite elongation [46]. These findings strongly suggest that JNK elicits neuroregenerative function as well, tempting us to think whether the anti-JNK strategy prevents spontaneous axonal regeneration after SCI. JNK inhibition by D-JNKI1 did not reduce long-term sprouting of the serotonergic fibers in the glial scar after SCI [45]. In this case, the single administration of D-JNKI1 was performed 6 h after SCI, under the experimental condition in which D-JNKI1 might not affect the relatively late-onset sprouting. On the other hand, continuous administration with SP600125 did not interfere with axonal branches extending from the CST into the gray matter far rostral to the lesion and, more likely, preserved the axonal branches sprouting in the rostral part close to the lesion epicenter more frequently compared with vehicle-treated control mice [44]. The reason why SP600125 efficiently leads to axonal regeneration after SCI is not clear. As a plausible explanation, SCI-induced JNK activation under the axonal degeneration process may be stronger than that under the axonal regeneration process, because a significant activation of JNK in the SC is observed at 1–3 days post-injury [44]. However, further investigation as to whether JNK inhibition especially during the axonal regeneration process worsens functional recovery after SCI is informative in considering the adverse effect of JNK inhibitors.

Taken together, JNK activity (at least in neurons and oligodendrocytes) can contribute to the pathogenesis of SCI. In addition, several reports have described that JNK activity in endothelial cells and astrocytes may be involved in the breakdown of the blood–CSF barrier and neuropathic pain, respectively, after SCI [45,47]. Although JNK is closely related to innate and adaptive immunity in various pathological situations, neither activation of microglial cells nor infiltration of myeloperoxidase-positive neutrophils were affected under the condition in which JNK inhibitors improved locomotor recovery after SCI [5,45,48]. Further precise studies are required, but JNK may not primarily orchestrate inflammatory events in the secondary damage of SCI.

## 4. Involvement of p38 in the Pathogenesis of SCI

### 4.1. p38 as a Central Player in Inflammatory Responses

In the secondary phase of SCI, infiltrated leukocytes and activated glial cells exacerbate tissue damage by releasing proinflammatory cytokines/chemokines, proteases, and reactive oxygen intermediates, though they exhibit certain beneficial aspects as well in some cases. The post-traumatic inflammatory responses may contribute to axonal degeneration, neuronal and oligodendrocytic cell death, and scar formation, and finally result in the impairment of neuronal function [49]. A number of studies have described that proinflammatory cytokines such as IL-6, TNF-α and IL-1β are potent mediators for the pathogenesis of SCI at the early stage of secondary damage [50]. Initially, p38 has been identified as a target protein for cytokine-suppressive anti-inflammatory drugs (CSAIDs) as well as a lipopolysaccharide (LPS)/TLR-activated kinase and shown to contribute to mRNA stabilization of IL-1β and TNF-α [51,52]. In addition to the function of transducing extracellular signals to the transcriptional machinery through regulating transcriptional factors (e.g., activating transcription factor 2 (ATF2), myocyte-specific enhance factor 2C/A, cAMP response element binding protein (CREB) and CEBP-homologous protein) as substrates of p38, p38 has been found to play an important role in both translation and stability of inflammatory mRNAs. For instance, AU-rich elements (AREs) found in the 3′-untranslated region of TNF-α mRNAs are binding sites for various factors regulating mRNA decay. The downstream kinase of p38, MAPK-activated protein kinase 2 (MK2 also known as MAPKAPK2), interferes with the interaction between AREs and the binding factors and thereby stabilizes TNF-α mRNA [53]. Expression of IL-6, which provokes activation and infiltration of leukocytes as one of its pleiotropic functions in SCI, is controlled by the mechanism of p38/MK2-induced mRNA stabilization as well [54]. Therefore, p38 can mobilize major SCI-related proinflammatory cytokines in the post-traumatic inflammatory process.

Using genetically manipulated mice and p38 inhibitors, it has been well documented that p38 plays important roles in various steps of inflammatory responses as follows: (i) p38 in macrophages stimulated with different TLR ligands regulates the expression of proinflammatory factors (e.g., inducible nitric oxide synthase (iNOS), cyclooxygenase 2, IL-6 and TNF-α) via gene regulatory mechanisms at both transcriptional and post-transcriptional levels [5,55]; (ii) p38 is required for the LPS-, TNF-, or UV-B-induced maturation of monocyte-derived dendritic cells (DCs) [5]; (iii) p38 is involved in production of interferon (IFN)-γ and Th1 differentiation of CD4^+^ T cells under stimulation with antigen and/or cytokines [5,56]; (iv) p38 upregulates the translational regulation of cytokine production in NKT cells through activating the MAPK-interacting serine/threonine kinase (Mnk)-eukaryotic translation initiation factor 4E (eIF4E) pathway, a downstream target of p38 [57]; (v) p38 is involved in DC-driven Th17 differentiation, Th17 proliferation, and regulation of IL-17 expression in Th17 [33,34]. As for IL-17 expression in Th17 cells, both the transcriptional regulation via ATF2/CREB activated by p38α and the translational regulation via the p38α/Mnk-eIF4E pathway have been proposed [35,36]. In conjunction with the fact that inhibition of p38 efficiently blocks the highly pathogenic avian influenza virus (HPAIV)-induced “cytokine storm”, the p38 pathway can regulate inflammatory responses as a central player [58].

### 4.2. Spatial Activation of p38 after SCI

In addition to neurons and oligodendrocytes, activation of p38 was observed in activated microglia/macrophages, infiltrated neutrophils, and reactive astrocytes forming a glial scar after SCI [39,40,59,60]. Several reports have described the mechanism of p38-mediated SCI development. In a complete transection model of SCI, activation of p38 induced the expression of iNOS in activated microglia/macrophages and then caused a decrease in NeuN^+^ cells. The loss of neuronal cells was effectively inhibited by either a p38 inhibitor, SB203580, or N(ω)-nitro-l-arginine methyl ester, suggesting that the p38/NO signaling axis mediates the microglial cell-induced neurotoxicity after SCI [59]. In the SC after contusion injury, induction of IL-1β expression and p38 phosphorylation was observed prior to neuronal apoptosis identified as the TUNEL^+^/active caspase-3^+^ somas in the gray matter. An inhibitor of the IL-1β pathway, IL-1Ra suppressed phosphorylation of p38, indicating that p38 functions downstream of IL-1R1. Likewise, the neuronal apoptosis was sensitive to both IL-1Ra and SB203580, suggesting that the IL-1β/p38 signaling axis is involved in neuronal apoptosis after SCI [61].

### 4.3. Correlation between p38 Pathway Inhibition and Functional Recovery after SCI

#### 4.3.1. p38 Inhibitors

Initially, SB203580 was reported to prevent damage to hindlimb function after SCI [62]. In contrast, a subsequent study has shown that SB203580 could not improve functional recovery after SCI [60]. The two reports are controversial in spite of employing a similar contusion SCI model and the same administration protocol (continuous intrathecal administration). The difference in intensity of SC damage between the two cases might influence the beneficial effect of p38 inhibition on SCI. In the former report, moreover, the treatment with SB203580 did not affect inflammatory responses in the lesion area, indicating that the anti-inflammatory potential of p38 inhibition may not work as a primary therapeutic effect on SCI.

Then, we have recently validated whether p38α is a potential therapeutic target in SCI. A single-copy disruption of *p38α* gene (p38α^+/−^, p38α^−/−^ is embryonic lethal as described above) reduced the tissue degenerative events (e.g., induction of various cytokines/chemokines, leukocytic infiltration, apoptosis of oligodendrocytes, loss of neuronal cells, and reactive astrogliosis) and augmented the tissue regenerative events (e.g., recruitment of oligodendrocyte progenitor cells, compaction of microglia/macrophages, and axonal regrowth), thereby causing a better functional recovery following lateral hemisection SCI. In our investigation, hence, genetic inhibition of p38α markedly suppressed at least the SCI-induced inflammatory responses: an increase in infiltration of both T lymphocytes and monocytes/macrophages into the lesion epicenter, and upregulation of inflammatory-related proteins (e.g., C-X-C motif chemokine 12, macrophage inflammatory protein (MIP)-1α, MIP-2, and matrix metalloproteinase 9) in the SC. These findings strongly suggest that the decrease of leukocytic infiltration associated with the suppression in expression of inflammatory-related proteins may contribute to the reduced development of SCI in p38α^+/−^ mice and further that inhibition of p38 can ameliorate SCI-associated inflammatory responses. In addition, the analysis using Texas Red-BDA, an anterograde tracer, showed that axonal regeneration after SCI was accelerated or enhanced in the caudal part of the SC in p38α^+/−^ mice compared with WT mice. Notably, SB239063, a p38 inhibitor that is transferable across the blood–CSF barrier, significantly improved functional recovery after SCI in WT mice if orally administered once a day at 1–3 days post-injury [63]. An overview of the SAPK-associated pathological outcome, especially the neuronal degenerative process in SCI, is shown in Figure 1B.

#### 4.3.2. Minocycline

Minocycline, known as a second-generation tetracycline, is an interesting candidate in the treatment of traumatic CNS injuries and neurodegenerative diseases because it possesses neuroprotective and anti-inflammatory properties. A number of reports have proposed that minocycline-evoked anti-inflammatory and anti-apoptotic actions could be mediated through inhibition of p38 as follows: (i) low doses of minocycline protected neurons in mixed spinal cord cultures from *N*-methyl-d aspartate excitotoxicity by inhibiting the p38-promoted microglial activation [64]; (ii) minocycline protected cerebellar granule neurons from NO-induced cell death via reduction of p38 activity [65]; (iii) minocycline ameliorated carrageenan-induced inflammation-associated hyperalgesia through inhibiting p38 activation in spinal microglia [66]. In fact, it has been shown that the minocycline-p38 inhibition loop improves functional recovery after SCI mainly by decreasing cell death of oligodendrocytes [67]. In that report, minocycline inhibited oligodendrocytic apoptosis via reduction of the p38 activity-dependent pro-nerve growth factor (proNGF) production in microglia, leading to the improvement of functional recovery after SCI.

NGF can promote cell death via stimulation of p75 neurotrophin receptor (p75^NTR^) in addition to the well-known function of neuronal differentiation/survival via tyrosine receptor kinase A (TrkA). Likewise, proNGF, the unprocessed NGF precursor, can bind p75^NTR^ preferentially over TrkA, and the expression of p75^NTR^ is specifically upregulated in oligodendrocytes after SCI. Thus, the proNGF/p75^NTR^ signaling axis-induced apoptosis may occur predominantly in oligodendrocytes after SCI [68].

In another report, gene array analysis of mRNA from the SCs of rats with SCI showed the expression of p38β2 was typically and specifically downregulated by treatment with minocycline, the phenomenon of which is of interest [69]. In regards to the LPS-induced neurotoxicity secondary to the proinflammatory cytokine production in microglia, however, p38β but not p38α is dispensable in the brain, which is similar to the case of peripheral inflammation mentioned above [24,70]. Furthermore, neuron-selective microRNAs (miRs)—miR-124 and miR-128—selectively deplete p38α but not p38β in neurons, which results in loss of function of the p38/Mnk-eIF4E pathway in neurons. Thus, p38β cannot compensate p38α loss at least in the translational machinery mediated by the Mnk-eIF4E pathway [71]. Further investigation is required for the pathophysiological significance of minocycline treatment-specific downregulation of p38β2.

Currently, an interventional clinical trial, MASC (Minocycline in Acute Spinal Cord Injury) in phase III, is in progress with minocycline for patients with SCI (clinicaltrials.gov, registration number NCT01828203). The indication expansion for minocycline is much expected [72].

#### 4.3.3. Plant-Derived Agents

Some plant-derived agents showing a beneficial effect on SCI can inhibit the p38 pathway. Curcumin, a yellow polyphenol derived from the *Curcuma longa* plant, is commonly used as a spice and also has a variety of medicinal properties including anti-inflammatory, analgesic, anti-oxidant, and antiseptic activity. It has been clearly demonstrated that curcumin enhances locomotor and sensory recovery after SCI at least partly through inhibiting the nuclear factor κ-light-chain-enhancer of activated B cells (NF-κB) pathway [73].

NF-κB elicits pleiotropic functions by regulating the transcription of various genes such as cytokines/chemokines, adhesion molecules, proinflammatory transcription factors, proinflammatory enzymes, and so on. In addition, it is well described that NF-κB is activated downstream of TLR and cytokine receptors like p38 is. In the CNS, the basal activity of NF-κB in glial cells is low but highly inducible in response to the change of neural environment, which may play a crucial role in brain inflammation [74]. In fact, transgenic mice with astrocyte-specific loss of function of NF-κB activity show a dramatic improvement of functional recovery after contusive SCI [75]. Therefore, NF-κB activity at least in glial cells contributes to the pathogenesis of SCI. Subsequently, it has been demonstrated that curcumin ameliorates secondary damage (e.g., production of IL-6, TNF-α, IL-1β, and nitric oxide) presumably through inhibiting both the TAK1/MKK6/p38 pathway and the NF-κB pathway, which may contribute to the improvement of locomotor recovery after SCI [76].

Geraniol (an acyclic monoterpene alcohol) is primarily found in rose oil, palmarosa oil, and citronella oil and commonly used in flavors and perfumes due to its rose-like scent. Geraniol has various properties including antibacterial, immune regulation, insecticidal, and antitumor activities. It has been demonstrated that Geraniol ameliorates secondary damage (e.g., edema, induction of proinflammatory cytokines, oxidative stress, and neural apoptosis) and improves locomotor recovery after SCI. As the main signaling mechanism of action, geraniol was shown to inhibit the SCI-upregulated expression of NF-κB and p38 [77]. Through a similar mechanism, improvement of locomotor recovery after SCI was observed in rats treated with asiaticoside, a terpenoid component extracted form *Centella asiatica* [78].

### 4.4. p38 as a Promising Target for Therapeutic Intervention in SCI?

Accumulating evidence with animal models suggests that p38 plays a crucial role in the pathogenesis of SCI and that p38 is expected as a clinical target for the treatment of SCI. However, p38 signaling has a wide spectrum of biological activities beyond the inflammatory/stress responses in the CNS [12]. In addition, the obvious resistance of p38α^+/−^ mice to various pathological inputs raises the question whether a full inhibition of p38 results in improvement of clinical symptoms [36,63,79,80,81,82]. This notion may be partly supported by the fact that highly concentrated application of SB203580 loses its beneficial effect on SCI [62]. Likewise, mitogen- and stress-activated kinases (MSKs) 1 and 2 that are activated downstream of p38 play important roles in macrophages as follows: production of the anti-inflammatory cytokine, IL-10; production of IL-1Ra; and an increase in transcription of dual-specificity protein phosphatase 1 (DUSP1 also known as MAPK phosphatase-1) leading to dephosphorylation/inactivation of p38. Thus, the p38/MSK signaling axis can function as the negative feedback loop in p38-mediated inflammatory responses [83]. Furthermore, p38 inhibitor-based therapeutic investigation is sometimes limited because of its specificity [84]. Therefore, further precise investigation is needed, focusing on the exact spatial and temporal activation of p38 which positively or negatively contributes to the functional outcome after SCI. As a result, p38 inhibition will be received as a therapeutic option without adverse effects.

## 5. Closing Remarks

Here, we gave an overview on roles of SAPKs in SCI and, in particular, discussed the potential of targeting the p38 pathway as a disease-modifying therapy in SCI. In addition to its role in the development of neuronal degeneration after SCI, p38 may contribute to SCI-associated neuropathic pain development and maintenance. After spinal nerve ligation (SNL) that is commonly used as a neuropathic pain model, p38 was markedly activated predominantly in spinal microglia, and its activity was maintained over 3 weeks [85]. The relationships between nerve-injury-induced microglial activation and pain sensitization are well characterized [86] Therefore, p38 may represent a potent clinical target for neuropathic pain management after SCI. In fact, the therapeutic effect of SB681323, a p38 inhibitor, on neuropathic pain following nerve trauma was studied in a past clinical trial (clinicaltrials.gov, registration number NCT00390845). It has been reported that nearly 80% of patients with SCI are seriously affected by pain and unpleasant sensations [87]. The development of emerging strategies for both functional recovery and neuropathic pain management after SCI is regarded as a clinically and socially urgent matter.

## Abbreviations

Aβ	Amyloid β
AD	Alzheimer’s disease
ARE	AU-rich element
ASK1	Apoptosis signal-regulating kinase 1
ATF2	Activating transcription factor 2
BDA	Biotinylated dextran amine
CNS	Central nervous system
CREB	cAMP response element binding protein
CSAID	Cytokine-suppressive anti-inflammatory drug
CSF	Cerebrospinal fluid
CST	Corticospinal tract
DCs	Dendritic cells
DLK	Dual leucine zipper-bearing kinase
EAE	Experimental autoimmune encephalomyelitis
eIF4E	Eukaryotic translation initiation factor 4E
HPAIV	Highly pathogenic avian influenza virus
IL	Interleukin
IFN-γ	Interferon-γ
iNOS	Inducible nitric oxide synthase
JNK	c-Jun *N*-terminal kinase
LPS	Lipopolysaccharide
MAPK	Mitogen-activated protein kinase
MAP2K	MAPK kinase
MAP3K	MAPKK kinase
Mcl-1	Myeloid cell leukemia sequence-1
miR	MicroRNA
MIP	Macrophage inflammatory protein
MK2	MAPK-activated protein kinase 2
MLK	Mixed lineage kinase
Mnk	MAPK-interacting serine/threonine kinase
MPTP	1-Methyl-4-phenyl-1,2,4,6-tetrahydropyridine
MSK	Mitogen- and stress-activated kinase
NF-κB	Nuclear factor κ-light-chain-enhancer of activated B cells
NGF	Nerve growth factor
p75^NTR^	p75 Neurotrophin receptor
SAPK	Stress-activated protein kinase
SCI	Spinal cord injury
SCG10	Superior cervical ganglion 10
TAB1	TAK1-binding protein 1
TAK1	TGF-β-activated kinase 1
TAO2	Thousand-and-one amino acid kinase 2
TGF-β	Transforming growth factor-β
Th	T-helper
TNF-α	Tumor necrosis factor-α
TNIK	TNF receptor-associated factor 2- and NCK-interacting protein kinase
TLR	Toll-like receptor
TPL2	Tumor progression locus 2
TrkA	Tyrosine receptor kinase A
TWY	Tiptoe-walking Yoshimura
UV	Ultraviolet
ZAP70	ζ chain-associated protein kinase
